# Influencing factors for delayed diagnosed injuries in multiple trauma patients – introducing the ‘Risk for Delayed Diagnoses Score’ (RIDD-Score)

**DOI:** 10.1007/s00068-024-02571-2

**Published:** 2024-06-26

**Authors:** Denis Gümbel, Gerrit Matthes, Axel Ekkernkamp, Fabian Laue, Rolf Lefering, The TraumaRegister DGU

**Affiliations:** 1 Department of Trauma, Reconstructive Surgery and Rehabilitation Medicine, University Medicine Greifswald, Ferdinand-Sauerbruch-Straße, 17475 Greifswald, Germany; 2 Department of Trauma and Orthopaedic Surgery, BG Klinikum Unfallkrankenhaus Berlin gGmbH, Warener Straße 7, 12683 Berlin, Germany; 3 Institute for Research in Operative Medicine, University Witten/Herdecke, Cologne, Germany; 4 Department of Trauma and Orthopaedic Surgery, Klinikum Ernst von Bergmann gGmbH, Charlottenstrasse 72, 14467 Potsdam, Germany

**Keywords:** Delayed diagnosed injury, Missed injury, Trauma, Multiple injuries, Trauma registry

## Abstract

**Purpose:**

Delayed diagnosed injuries (DDI) in severely injured patients are an essential problem faced by emergency staff. Aim of the current study was to analyse incidence and type of DDI in a large trauma cohort. Furthermore, factors predicting DDI were investigated to create a score to identify patients at risk for DDI.

**Methods:**

Multiply injured patients admitted between 2011 and 2020 and documented in the TraumaRegister DGU® were analysed. Primary admitted patients with severe injuries and/or intensive care who survived at least 24 h were included. The prevalence, type and severity of DDI were described. Through multivariate logistic regression analysis, risk factors for DDI were identified. Results were used to create a ‘Risk for Delayed Diagnoses’ (RIDD) score.

**Results:**

Of 99,754 multiply injured patients, 9,175 (9.2%) had 13,226 injuries first diagnosed on ICU. Most common DDI were head injuries (35.8%), extremity injuries (33.3%) and thoracic injuries (19.7%). Patients with DDI had a higher ISS, were more frequently unconscious, in shock, required more blood transfusions, and stayed longer on ICU and in hospital. Multivariate analysis identified seven factors indicating a higher risk for DDI (OR from 1.2 to 1.9). The sum of these factors gives the RIDD score, which expresses the individual risk for a DDI ranging from 3.6% (0 points) to 24.8% (6 + points).

**Conclusion:**

DDI are present in a sounding number of trauma patients. The reported results highlight the importance of a highly suspicious and thorough physical examination in the trauma room. The introduced RIDD score might help to identify patients at high risk for DDI. A tertiary survey should be implemented to minimise delayed diagnosed or even missed injuries.

## Introduction

Optimal diagnosis and treatment of patients with multiple injuries can be challenging, even for experienced staff. During initial Emergency Department (ED) management, rapid evaluation and prioritisation of potentially life-saving interventions and diagnostic procedures are crucial. This demands a structured priority-orientated approach to initial assessment and management, as described by ATLS® [1].

Despite a thoughtful, structured approach, including a thorough clinical examination, several studies confirm that significant injuries are being missed during management [2–6]. These missed injuries have also been termed ‘the trauma surgeon’s nemesis’ since undetected injuries have been shown to lead to significant morbidity and mortality among trauma patients. They can result in prolonged hospital stays, increased treatment costs, and medico-legal issues [2, 7, 8]. If missed injuries are detected during in-hospital treatment the term ‘delayed diagnosed injuries’ should better be used.

The etiology of missed injuries or delayed diagnoses is multifactorial. So far, identified influencing factors include the level of patient consciousness, time of presentation, experience of treating doctors, and efficacy in dealing with radiological imaging [9–13].

Also, the secondary survey may be interrupted by other priorities during resuscitation, i.e. immediate emergency operations leading to injuries missed during initial clinical evaluation [14].

The complexity of managing multiple injured patients has led to the concept of a tertiary assessment, as described by Enderson et al. The authors conclude that a routine ‘tertiary survey’ reduces the risk for undiagnosed injuries, improves patient care and may have favourable medicolegal implications [15–17].

The present study aimed to investigate the incidence of delayed diagnosed injuries in a large cohort of multiply injured patients. Moreover, we set out to identify and quantify factors increasing the risk of missing injuries during the survey to identify patients/situations under risk.

## Patients and methods

### TraumaRegister DGU®

The TraumaRegister DGU® (TR-DGU) of the German Trauma Society (Deutsche Gesellschaft für Unfallchirurgie, DGU) was founded in 1993. The aim of this multi-centre database is a pseudonymised and standardised documentation of severely injured patients.

Data are collected prospectively in four consecutive time phases from the site of the accident until discharge from hospital: (A) pre-hospital phase, (B) emergency room and initial surgery, (C) intensive care unit and (D) discharge. The documentation includes detailed information on demographics, injury pattern, comorbidities, pre- and in-hospital management, course on intensive care unit, relevant laboratory findings including data on transfusion and outcome of each individual. The inclusion criterion is admission to hospital via emergency room with subsequent ICU/IMC care or admis- sion with vital signs and death before admission to ICU.

The infrastructure for documentation, data management, and data analysis is provided by AUC - Academy for Trauma Surgery (AUC - Akademie der Unfallchirurgie GmbH), a company affiliated to the German Trauma Society. The scientific leadership is provided by the Committee on Emergency Medicine, Intensive Care and Trauma Management (Sektion NIS) of the German Trauma Society. The participating hospitals submit their data pseudonymised into a central database via a web-based application. Scientific data analysis is approved according to a peer-review procedure laid down in the publication guideline of TraumaRegister DGU®.

The participating hospitals are primarily located in Germany (90%), but a rising number of hospitals of other countries contribute data as well (at the moment from Austria, Belgium, China, Finland, Luxembourg, Slovenia, Switzerland, The Netherlands, and the United Arab Emirates). Currently, more than 28,000 cases from nearly 700 hospitals are entered into the database per year.

Participation in TraumaRegister DGU® is voluntary. For hospitals associated with TraumaNetzwerk DGU®, however, the entry of at least a basic data set is obligatory for reasons of quality assurance.

The present study is in line with the publication guidelines of the TraumaRegister DGU® and registered as TR- DGU project ID 2021-034.

### Patients

All data were extracted from the TraumaRegister DGU®. Datasets from January 2011 until December 2020 were analysed. Inclusion criteria were primary admitted patients, Maximum Abbreviated Injury Scale (MAIS) ≥ 3, admission to ICU, and survival > 24 h in the hospital, giving the treating team time to detect all injuries. Transferred patients from other hospitals and patients transferred to another hospital within a few hours were excluded. Patients were also excluded if their diagnoses were documented without information on the time of diagnosis. Analysed parameters included age, sex, Injury Severity Score (ISS), Abbreviated Injury Score (AIS) for head, face, neck, thorax, abdomen, spine, upper and lower extremity, Glasgow Coma Scale (GCS), blood transfusion, trauma mechanism, type of injury, hospital stay, ICU stay, days ventilated, mortality rate and diagnosed injury after admission on ICU marked in the web interface. Also, the trauma centre level of care (1 = supra-regional; 2 = regional; 3 = local) was assessed.

Delayed diagnosed injuries (DDI) were defined as injuries marked as diagnosed after admission to the ICU. This information is available in 84% of all diagnoses. Among such DDI, there may be some conditions that may have developed during the course of treatment, like an increasing intra-cranial bleeding or a late vessel rupture. The data collected in TR-DGU would not allow to separate those late events from those missed to be diagnosed in the ER. The DDIs were analysed in terms of severity (Abbreviated Injury Scale) and body region. A patient with at least one DDI was placed in the DDI patient group. A patient was excluded from the analysis if there was no information about the time of diagnosis for any diagnosis. If a time point was missing for a specific diagnosis but available for the other diagnoses of a case, this diagnosis was not considered DDI.

### Statistical analysis

The data were analysed using SPSS statistical software (version 26, IBM Inc., Armonk, NY, USA). Descriptive data are presented as number of cases with percentage, or as mean with standard deviation (SD), respectively. In case of a rather skewed distribution, the median with inter-quartile range (IQR) was presented instead of mean/SD. Formal statistical comparisons with test statistics were avoided due to the large sample size. With thousands of patients in each group, even minimal differences (like +/- 1% for categorical variables) would be statistically significant. Therefore, the relevance of the observed difference should primarily be considered.

A multivariate logistic regression analysis was performed to identify patients at risk for a delayed diagnosis. The following independent predictors were included: blunt/penetrating mechanism; the number of diagnoses; polytrauma [18]; unconsciousness (GCS 3–8), blood transfusion; emergency surgery; transfer from emergency room to operation theatre; admission during the night; admission during the weekend; Hospital level of care. Results were presented as odds ratios (OR) with 95% confidence intervals. Based on these results, a simplified point score was created, summarising the individual risk for DDI.

The following interventions were counted as emergency interventions: brain decompression/drainage, thoracotomy, laparotomy, revascularisation, embolisation, and external stabilisation of the pelvis or extremities.

## Results

### Study cohort

**Fig. 1:**
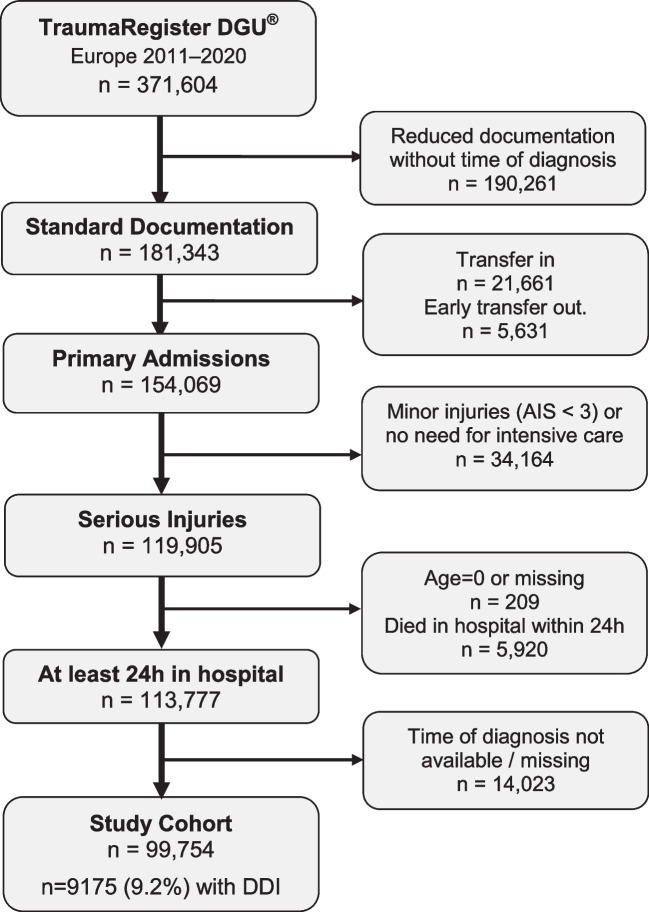
Selection of patients

The study cohort comprised 99,754 patients who met the inclusion criteria (Fig. 1). The mean age was 49 years, and the majority were males (71%). The average ISS was 19.1 points, and 82% were treated in a Level 1 trauma centre.

Patients had a total number of 494,990 diagnoses documented. The median number of diagnoses per case was 4 (IQR 3–6). Most diagnoses were identified in the emergency room (92.2%), in 5.1% of diagnoses the time point was missing, and 13,226 diagnoses (2.7%) were coded as being first identified after ICU admission (DDI). These 13,226 diagnoses were found in 9,175 of the 99,754 patients (9.2%).

Patients with and without a DDI were compared in Table 1. Patients with DDI were more seriously injured (ISS 25.0 versus 18.5), had more injuries (median number 6 versus 4), were more often unconscious (25.1% versus 17.0%), and suffered more often from severe head injury (49.2% versus 36.2%). Car passengers and motorbike riders were more frequently found in the DDI group. The whole-body computed tomography (WB-CT) rate was similar in both groups (83.7% versus 85.0%). The date (weekday/weekend), as well as the time of admission (day/night), also did not show a relevant difference. According to the higher injury severity, hospital and ICU length of stay was longer in DDI patients as well as a higher hospital mortality rate: 10.8% in patients with DDI, and 6.5% patients without DDI.

**Table 1:** Characteristics of patients with and without delayed diagnosed injuries (DDI)

	Patients without DDI *n* = 90,579	Patients with DDI *n* = 9,175
Age (years (SD))	49.5 (22.0)	48.2 (21.9)
Sex (male %)	71.1	71.7
ISS (points (SD)	18.5 (11.2)	25.0 (13.4)
New ISS (points (SD)	23.2 (14.0)	32.0 (16.4)
Number of diagnoses*	4 [3-6]	6 [4-9]
Polytrauma (Berlin definition [18], %)	16.3	30.4
Severe head injury (AIS 3+) (%)	36.2	49.2
Unconscious (GCS 3–8, %)	17.0	25.1
Blunt trauma (%)	95.7	97.4
Cause of accident (%)		
Car	20.9	25.7
Motorbike	12.8	16.0
Bicycle	10.2	9.4
Pedestrian	6.5	7.0
High fall > 3 m	16.6	15.3
Low fall < 3 m	21.2	17.4
Trauma center level of care (%)		
Level 1	82.5	79.3
Level 2	14.3	16.6
Level 3	3.1	4.2
Whole-body CT (%)	83.7	85.0
Admission during night time (%)	38.2	39.0
Admission during weekend (%)	45.2	45.4
Shock in ED (BP ≤ 90 mmHg) (%)	12.6	18.7
Blood transfusion in ED (%)	8.7	15.7
Emergency surgery (%)	23.5	29.3
Hospital mortality beyond 24 h (%)	6.5 (*n* = 5883)	10.8 (*n* = 995)
Length of stay on ICU (days)*	3 [3-8]	6 [2-16]
Length of ventilation (days)*	0 [0-2]	1 [0-9]
Length of stay in hospital (days)*	13 [7-22]	18 [10-30]

*results presented as median with quartiles

In this study cohort, 82.2% of patients were treated at a level 1 trauma centre, 14.5% at level 2, and 3.2% at level 3. In the DDI group, 79.3% of patients were treated at a level 1 trauma centre, 16.6% at level 2, and 4.2% at level 3, indicating a higher proportion of patients with DDI in level 2 and 3 hospitals.

### Delayed diagnosed injuries by body region

**Fig. 2:**
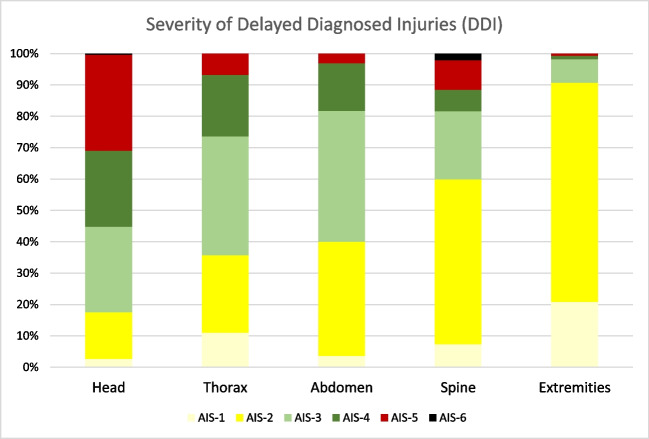
The severity of DDI injuries varies by body region. If multiple injuries occur in the same body region, the worst one is considered. The number of patients per body region ranges from 865 (spine) to 3287 (head)

At a patient level, the most frequently affected body region was the head (*n* = 3,287; 3.3% of all cases, 35.8% in DDI cases), followed by the extremities (*n* = 3051, 3.1% of all cases / 33.3% of DDI cases). DDI in the thorax were found in 1811 cases (1.8% / 19.7%), and 1018 patients had a DDI in the abdomen (1.0% / 11.1%). Spinal injuries diagnosed lately were found in 865 patients (0.9% / 9.4%). The injury severity of DDI varied among the body regions, as shown in Fig. 2.

### Risk factors for DDI

Based on the univariate findings (Table 1), a logistic regression analysis was performed with DDI as the dependent variable. The predictors listed in Table 2 were found to be associated with an increased risk of DDI. Predictors that did not reach an odds ratio above 1.20 were removed from the model. All predictors could be measured, or at least estimated, in the ED.

**Table 2:** Results from logistic regression analysis with DDI as dependent variable (overall prevalence: 9.2%)

Risk factor	Prevalence of risk factor	Prevalence of DDI if risk factor present	Odds Ratio (OR)	95% CI for OR
2 + body regions affected*	65.0%	11.3%	1.88	1.78–1.99
Unconsciousness (GCS 3–8)	20.2%	13.6%	1.72	1.64–1.81
Small (level 3) and medium-sized (level 2) trauma centers	17.8%	10.7%	1.46	1.38–1.54
Blood transfusion required before ICU admission	9.6%	15.5%	1.45	1.36–1.55
Cardiac arrest / CPR, pre-hospital or in the ED	2.2%	16.7%	1.34	1.19–1.51
Motorbike rider	12.9%	11.2%	1.34	1.26–1.43
Car passenger	21.1%	11.1%	1.31	1.25–1.38
6 or more injuries identified in the ED	32.5%	12.8%	1.27	1.21–1.33

Predictors with odds ratio (OR) < 1.20 were removed from the final model; the remaining predictors are listed with decreasing OR

*Body regions according to the first digit of AIS: head, face, neck, thorax, abdomen, upper extremity, lower extremity, soft tissue. Minor injuries (AIS 1) were disregarded

**Fig. 3:**
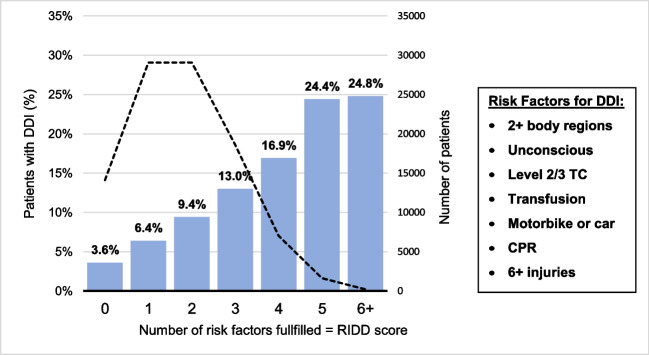
Risk for Delayed Diagnoses Score (RIDD-Score): Prevalence of DDI per number of risk factors fulfilled. The dotted line represents the number of patients with the respective number of risk factors. TC = trauma center; CPR = cardio-pulmonary resuscitation

The RIsk for Delayed Diagnoses score (RIDD score) is calculated by adding one point for each risk factor present. This results in a score ranging from 0 to 7 where the theoretical maximum value (7 points) was observed in only 5 cases. The prevalence of DDI increased with RIDD and ranges from 3.6% (0 points) to 24.8% (6 + points) (Fig. 3).

## Discussion

Missed and delayed diagnosed injuries (DDI) have been shown to affect trauma patients’ morbidity and mortality [2, 3]. Understanding of the underlying etiology is crucial for minimising incidence rate in multiply injured patients. Furthermore, identifying circumstances in which DDI are more likely to occur helps to raise suspicion in the treating trauma team.

To investigate undetected injuries, it is of pivotal importance to first consent a definition of missed and delayed diagnosed injuries.

Missed injuries (MI) refer to injuries that have not been detected throughout the entire in-hospital treatment. In contrast, delayed diagnosed injuries (DDI) have been detected during hospital stay, but delayed, meaning not at the time they could have been detected. DDI may therefore impact treatment during initial hospital stay. During primary survey in the ED the emergency team needs to identify life-threatening injuries first. During secondary survey, all injuries should be identified through a thorough examination by the trauma team with high suspicion. In this study, injuries first diagnosed in ICU are defined as ‘delayed diagnosed injuries’.

### Incidence of DDI

In this study, delayed-diagnosed injuries (DDI) were evaluated in the largest cohort of multiple trauma patients. DDI occurred in 9.2% of patients. 35.8% of delayed-diagnosed head injuries, 19.7% of thoracic injuries, and 33.3% of extremity injuries were found. 11.1% had abdominal, and 9.4% had delayed-diagnosed spine injuries.

Published incidence rates of DDI vary largely in the literature depending on the definition and study cohort. Incidence rates from 1.3% up to 25.8% have been reported in studies with limited sample sizes [2–5, 19–23]. In 1991, Laasonen et al. investigated missed injuries in 340 patients and identified 8.5% of these patients [21]. Similar results have been published by Buduhan et al. in 2000. The study group reports that 8.1% of 567 patients have missed injuries [2]. Vles et al. found missed injuries in only 1.3% of 3,879 patients in a prospective study published in 2003 [19]. In contrast, Brooks et al. published one year later, in 2004, a delayed injury rate of 22.2% in severely injured patients [3]. In 2011, Lawson et al. published an incidence rate of 9% in a larger cohort of 26,264 patients. This incidence rate is in line with our results. Reasons for differences in reported rates of MI and DDI beside unequal definitions and study cohorts are improving standards and procedures in the ED. They have contributed to reduce the rate of DDI over the last decades. For example, multislice computed tomography (MSCT), which is now a standard diagnostic modality in polytrauma patients, was not available to this extent 20 years ago. Also, implementation of standard course formats like ATLS® and emergency room trainings might have improved skills and expertise among trauma doctors worldwide [24]. The relatively high number of patients with delayed diagnosed head injuries raises the question of whether routine follow-up CT scans, especially in unconscious patients, may close a diagnostic gap since subdural hematomas may develop within 24 h to 48 h after trauma. Furthermore, following a high-energy trauma abdominal CT scan after 12 h to 24 h hours may reveal bowel or splenic injury that remained undetected on initial CT scans even when reviewed retrospectively.

Also, available tools like artificial intelligence (AI) may help to reduce DDI rates [25].

Finally, one has to keep in mind that a certain number of lesions (e.g., cerebral edema) may develop during the clinical course. Therefore, they might be diagnosed after the initial workup, leading to a misdocumentation as DDI. In a registry, those diagnoses cannot be identified reliably ex post.

Finally, one has to consider that some lesions addressed as DDI might also result from early interventions during initial treatment. For example, a pneumothorax might be caused by introducing a central venous line to the vena subclavia. Those entities are not to be identified by registry records.

As stated initially, the trauma team’s experience plays an important role in treating severely injured patients. Although individual training of team members is mandatory, monitored team performance depends on team training. Given a greater number of severely injured patients, teams in level I trauma centres may have more training, and management includes the identification of frequently missed injuries. This may also apply for ICU staff and may have led to a higher proportion of patients with DDI treated in level-2 and 3 hospitals.

How different hospital levels and patient numbers can affect the incidence of DDI and how this can be improved, i.e. team training, should be subject to further analysis.

### Factors influencing DDI

While treating multiple injured patients in the ED, the reported mechanism of injury gives valuable information on associated injuries. In car accidents, injuries to the thorax and lower extremities are frequently observed. In contrast, patients sustaining a fall from a height greater than 3 m have a higher chance of sustaining injuries to the lower extremities and spine [26, 27].

Overall, patients with DDI were more likely to be hypotensive at initial presentation (12.6% vs. 18.7%), showed a higher transfusion rate (8.7% vs. 15.7) and were more likely to be unconscious (17.0% vs. 25.1%). Due to the higher injury severity in DDI patients (ISS 25.0 versus 18.5), emergency surgery was significantly more often performed in patients with DDI (29.3% vs. 23.5%). Head injuries were much more prevalent in DDI cases. However, in some of these cases, intra-cranial bleeding may have progressed so that these injuries were not apparent in the initial scans. We did not find a correlation between age and incidence of DDI.

Possibly, DDIs occur due to a prioritised intervention in the ED before finishing a proper secondary survey following the paradigm ‘Treat first what kills first’. Undetected injuries may arise since resuscitation of severely injured patients has priority over complete identification of all injuries, especially in patients ‘in extremis’. Doing so, minor injuries might be identified during the ‘tertiary survey’ after resuscitation in the ICU. Even injuries that develop with latency contribute to a higher number of DDI.

The concept of a tertiary survey within 24 h has been shown to reduce the incidence of DDI [28]. The tertiary survey is a complete and systematic patient reevaluation, including primary and secondary surveys and a review of radiographs usually performed in the ICU after initial resuscitation. However, in a prospective study by Keijzers et al. evaluating a formalised tertiary survey, the authors did not find a significant reduction of missed injuries. Still, they reported a high number of injuries not detected within the initial hospital stay. Especially in patients with neurologic compromise, clinical examination can be challenging.

Finally, the value of the initial radiological workup needs to be discussed.

However, in advance, we have demonstrated that initial whole-body computed tomography is highly specific but has variable sensitivity for the detection of injuries in patients with suspected blunt trauma. In this context, we found that the best balance between sensitivity and specificity was achieved when the WBCT was performed about 30 min after admission [20].

#### The Risk for Delayed Diagnoses Score (RIDD-Score)

Within the presented study we were able to figure out a score estimating the risk of a DDI of an individual patient. This simple to be used instrument helps identify patients under risk already at the very beginning of the initial emergency treatment. However, in reverse, this does not mean that patients with a low score should be managed less thoroughly initially (Fig. 3).

### Limitations

Several factors may limit the results of this study and must be interpreted carefully. In addition to a possible selection error, register data are generally less valid than data provided by prospective randomized controlled studies.

Furthermore, DDI are defined as injuries first observed on ICU as diagnoses of patients in the TraumaRegister DGU® can only be differentiated between injuries identified in the ED and injuries first diagnosed on ICU. Furthermore, injuries missed until discharge from the hospital are not documented. The time point of diagnosis was not documented in all cases, and assumably, not all DDI were documented in the TraumaRegister DGU® as it requires active marking in the web-based interface.

Also, diagnoses in the TraumaRegister DGU® are documented as AIS codes. This may limit accuracy of delayed diagnoses as no exact code exists for each injury type.

Further studies need to investigate outcome of delayed diagnosed injuries.

## Conclusion

The study investigated delayed diagnosed injuries in a large trauma cohort of 99,754 multiple trauma patients with an incidence of delayed diagnoses of 9.2%.

A higher proportion of patients with DDI was treated in level 2 and 3 trauma centres. Thorax and the extremities were the most affected body regions. Overall, patients with DDI were more likely to be hypotensive at initial presentation (12.6% vs. 18.7%), showed a higher transfusion rate (8.7% vs. 15.7) and were more likely to be unconscious (17.0% vs. 25.1%). Interestingly, patients with DDI were more likely to be in shock (12.6% vs. 18.7%) and in a coagulopathic state (11.5% vs. 15.5%). Emergency surgery was significantly more often performed in patients with DDI than in the non-DDI group (23.5% vs. 29.3%). 41% of patients without DDI had combination injuries including a severe head trauma while 52.9% patients with DDI. The reported results highlight the importance of a highly suspicious thorough physical examination during secondary survey in the trauma room. Repeated clinical assessment during resuscitation should be implemented to minimise delayed diagnosed injuries. Clinical examination in the ED should be performed with high suspicion, and a tertiary survey is recommended. Especially in patients with a high RIDD-Score score (4 or higher), the treating trauma team must be alert in identifying all injuries in the ED, and a tertiary survey should be mandatory. Repated CT-scans may be necessary.

## Data Availability

No datasets were generated or analysed during the current study.
